# Performances of ventilator at simulated altitude

**DOI:** 10.1186/cc11083

**Published:** 2012-03-20

**Authors:** E Forsans, L Franck, T Leclerc, M Bensalah, J Tourtier, Y Auroy, C Bourrilhon

**Affiliations:** 1 HIA Val-de-Grâce, Paris, France; 2 Institut de Recherche Biomédicale des Armées, Brétigny sur Orges, France

## Introduction

We have assessed the ability of three ventilators to deliver to a normal lung model a set tidal volume (V_t_) at different simulated cabin altitudes. We studied the performance of the LTV-1200 (Viasys Healthcare, USA), the Elisée 350 (Resmed, Australia) and the Medumat transport (Weinmann, Germany).

## Methods

We used a decompression chamber to mimic the hypobaric environment at a range of simulated cabin altitudes of 2,438 and 3,657 m (8,000 and 12,000 feet). Ventilators were tested with a set fraction of inspired oxygen of 50% and V_t _set at 450. Respiratory rate was 12 breaths/minute. Comparisons of preset to actual measured values were accomplished using a *t *test for each altitude. The protocol included 36 measurements for each V_t _set at each simulated altitude. A significant difference was defined by *P *< 0.05.

## Results

**Figure 1:**
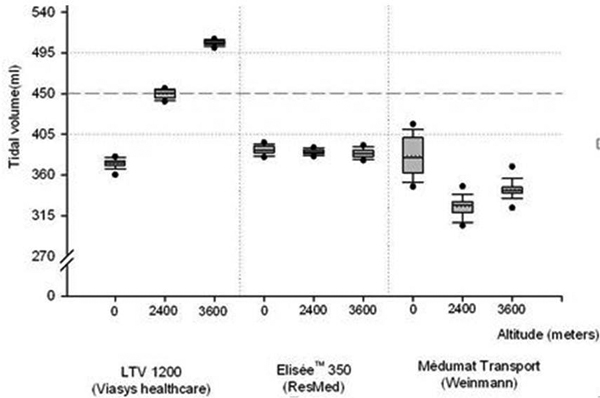


Figure 1 summarizes the data. Comparisons of actual delivered V_t _in altitude and set V_t _demonstrated a significant difference for the three ventilators.

## Conclusion

The LTV-1200 showed a very significant increase in V_t _delivered with increasing altitude (suggesting a lack of efficacy of altimetric correction in hypobaric conditions), whereas the Elisée 350 and Medumat transport delivered respectively a stable and a rather stable V_t_.

